# An IMM-Aided ZUPT Methodology for an INS/DVL Integrated Navigation System

**DOI:** 10.3390/s17092030

**Published:** 2017-09-05

**Authors:** Yiqing Yao, Xiaosu Xu, Xiang Xu

**Affiliations:** Key Laboratory of Micro-Inertial Instrument and Advanced Navigation Technology, Ministry of Education, School of Instrument Science and Engineering, Southeast University, Nanjing 210096, China; yucia@sina.com (Y.Y.); xuxiang@seu.edu.cn (X.X.)

**Keywords:** INS/DVL integrated navigation system, IMM, dynamic ZUPT, underwater navigation

## Abstract

Inertial navigation system (INS)/Doppler velocity log (DVL) integration is the most common navigation solution for underwater vehicles. Due to the complex underwater environment, the velocity information provided by DVL always contains some errors. To improve navigation accuracy, zero velocity update (ZUPT) technology is considered, which is an effective algorithm for land vehicles to mitigate the navigation error during the pure INS mode. However, in contrast to ground vehicles, the ZUPT solution cannot be used directly for underwater vehicles because of the existence of the water current. In order to leverage the strengths of the ZUPT method and the INS/DVL solution, an interactive multiple model (IMM)-aided ZUPT methodology for the INS/DVL-integrated underwater navigation system is proposed. Both the INS/DVL and INS/ZUPT models are constructed and operated in parallel, with weights calculated according to their innovations and innovation covariance matrices. Simulations are conducted to evaluate the proposed algorithm. The results indicate that the IMM-aided ZUPT solution outperforms both the INS/DVL solution and the INS/ZUPT solution in the underwater environment, which can properly distinguish between the ZUPT and non-ZUPT conditions. In addition, during DVL outage, the effectiveness of the proposed algorithm is also verified.

## 1. Introduction

Currently, precise positioning and navigation technology still remains a challenge for underwater vehicles due to blockages of the global positioning system (GPS). With complementary characteristics, the inertial navigation system (INS) and Doppler velocity log (DVL) are the most common navigation solutions for accomplishing underwater positioning tasks [1]. As a self-contained system, the INS is able to maintain high accuracy in short periods. However, its navigation errors accumulate with time [2]. To mitigate the degradation, the DVL is always adopted to provide the velocity information, which is integrated with INS by a Kalman filter (KF) [3].

There are various errors in the INS/DVL integrated navigation system, such as installation error and scale factor error [4,5]. Normally, the former one can be precisely calibrated off line. Once fixed, the installation error between INS and DVL changes little, which can be easily compensated. The in situ calibration can also be conducted with or without additional external information, such as that from GPS and acoustic navigation systems [6,7]. The scale factor error can also be estimated during the voyages [8]. However, as the scale factor varies with temperature, water density and salinity, there are always residual errors in the DVL velocity data, which are difficult to exclude further in the complex underwater environment [9].

In order to reduce the negative effect caused by the DVL velocity error, in this work zero velocity update (ZUPT) methodology is introduced to the INS/DVL-integrated navigation system to restrict the vehicle’s lateral velocity error. ZUPT is one of the most popular solutions in land vehicles navigation systems, and well utilizes the characteristics of the land vehicle motion. Static ZUPT was first raised to utilize the zero velocity condition during every stop to control the navigation error growth. The curve fitting and KF are two main methods for applying the static ZUPT, while the latter shows a higher degree of navigation accuracy [10]. When the KF-based ZUPT solution is employed, not only the velocity error of the vehicle, but also the attitude and positioning errors can be reduced. Besides, in order to further decrease the navigation errors even when the vehicle is moving, dynamic ZUPT was proposed [11,12]. According to the vehicle’s dynamic characteristics, when the vehicle does not jump off or slide on the ground, its velocity is expected to be in the forward direction. The zero velocity restrictions on the lateral and vertical directions will help restrain the divergence of the positioning error in the two directions [13,14]. In recent years, ZUPT methodology has also been widely utilized in the pedestrian navigation system, where external navigation information is unavailable [15,16]. In all the above applications, the ZUPT solution is used to mitigate the accumulative navigation error in the pure INS mode when the GPS and other navigation data is missing. When the external information is regained, the whole system will switch into the integration mode, where the ZUPT method becomes useless.

However, for the INS/DVL-integrated navigation system, where errors can be easily involved in the DVL velocity information due to the complex underwater environment, the ZUPT solution should be further explored to mitigate the lateral velocity and positioning errors caused by the DVL velocity errors. When the water current remains still or the underwater vehicle travels along the current, the lateral velocity of the vehicle should always be zero. Using the lateral constraint to replace the observation vector calculated by the DVL, the navigation performance can be improved because a more accurate observation vector can be constructed to restrict the accumulation of the positioning error.

To employ the ZUPT technology properly, its zero velocity condition needs to be correctly identified. In most research, the zero velocity condition is indicated by the inertial measurement units (IMUs). The mean value and standard deviation of IMUs during the static period are calculated to provide the threshold to judge the zero velocity condition [17]. An adaptive ZUPT algorithm using a sliding time window is presented to guarantee the zero velocity condition [18]. Fault detection methods are also used to identify the zero and non-zero velocity conditions [19]. However, the thresholds among these algorithms need to be carefully selected according to their IMU performance and the environment. Moreover, some detection methods show the characteristic of time delay, which would undermine the advantages of the ZUPT solution. For land vehicles, dynamic ZUPT always works because the carrier seldom jumps off or slides on the ground, and the observation vectors during the non-ZUPT periods can be regarded as noises. However, for the underwater vehicles, where the water current often exists during the voyage, the detection of the ZUPT condition is of great importance. Thus, it is critical to develop some new techniques to ensure a smooth switching process between the ZUPT and non-ZUPT modes for the INS/DVL-integrated navigation system.

Thus, to balance the dynamic ZUPT methodology and INS/DVL integration simultaneously, and to identify the ZUPT condition to realize the smooth switching process properly, an interactive multiple model (IMM)-aided ZUPT methodology for INS/DVL-integrated navigation system is proposed. Two integration models, which are denoted as the INS/ZUPT model and INS/DVL model, are established and updated simultaneously to represent different driving statuses. Given the Markov chain process for the transition between different models, the IMM algorithm can easily identify the current driving status and make a trade-off between the estimation performances of the two models according to their innovations and innovation covariance matrices. Compared to the traditional single INS/DVL model-based solution, the IMM-aided algorithm makes it possible to employ ZUPT methodology to deal with the zero velocity condition and the INS/DVL method to deal with the non-zero velocity condition, which may improve the navigation performance when the underwater vehicle travels under various patterns. Moreover, the zero velocity detector is no longer needed to identify whether the whole system can be operated under the ZUPT conditions or not, as it is self-contained in the IMM model probability update process.

The IMM algorithm was first widely applied in the target tracking area. To better predict the position of the target, the constant location (CL), constant velocity (CV), constant acceleration (CA) and constant turn rate (CT) models are fused by IMM to cover all of the possible motions [20,21]. In recent years, IMM has become popular in high-precision INS/GPS-integrated navigation systems. With the IMM method, the uncertainty of the KF parameters can be solved, where the process noise and observation noise can be covered by multiple models [22,23]. In this work, the ZUPT model is integrated with the INS/DVL model by IMM for the first time to well utilize the dynamic ZUPT condition in an integrated navigation system, rather than in a pure INS. This is the main contribution of the proposed method when compared to previous works. The IMM itself can smoothly switch between different models without other complex identification and switching algorithms. Meanwhile, when DVL data is unavailable, the proposed IMM-aided ZUPT methodology can continuously denote the status of the vehicle, which gives an indication of whether the system should be operated under the ZUPT algorithm, or under pure INS mechanization where no extra ZUPT detector module is required.

The rest of this paper is organized as follows. Section 2 gives an introduction of the ZUPT solution for the INS/DVL-integrated navigation system. Section 3 illustrates the IMM-aided ZUPT methodology. Simulations are shown in Section 4 and conclusions are drawn in Section 5.

## 2. ZUPT for INS/DVL-Integrated Navigation System

In this work, the dynamic ZUPT is explored, which provides the lateral and vertical velocity restrictions for the moving vehicles. When the water current remains still, it is highly possible that the underwater vehicle travels along the body-frame-forward direction, which is similar to land vehicles. Although DVL scale factor error can be estimated by INS/DVL integration, there are still errors left due to the complex underwater environment. The velocity information provided by DVL may contain biases, which would contaminate the navigation accuracy of the system. However, as the underwater vehicles are likely to be under simple motion for the most of the time, dynamic ZUPT is a very practical and effective method for reducing the accumulation of the lateral and vertical positioning errors. In this section, INS mechanization is described and its error model is derived. Then, INS/ZUPT is integrated by KF, where zero velocity in lateral and vertical directions of the vehicle’s body frame is introduced to the integration mechanization.

### 2.1. INS Mechanization and Its Error Model

First, define the coordinate frames employed in this work: *i*-frame: Earth-centered initially-fixed orthogonal reference frame; n-frame: Orthogonal reference frame aligned with the east–north–up (ENU) geodetic axes; *b*-frame: Orthogonal reference frame aligned with the inertial measurement unit (IMU) axes; $n^{\prime }$-frame: Calculated n-frame with small misalignment errors.

Taking the local geographical frame as the navigation frame, the differential equations of the attitude, velocity and position are [24]:

(1) $${\dot{C}}_{b}^{n}=C_{b}^{n}\left(\omega_{nb}^{b}\times \right)$$

(2) $${\dot{V}}^{n}=C_{b}^{n}f^{b}-\left(2\omega_{ie}^{n}+\omega_{en}^{n}\right)\times V^{n}+G^{n}$$

(3) $$\dot{L}=V_{N}/R_{M}$$

(4) $$\dot{\lambda }=V_{E}/\left(R_{N}\cos L\right)\;$$

(5) $$\dot{h}=V_{U}\;$$

where

(6) $$\omega_{nb}^{b}=\omega_{ib}^{b}-C_{n}^{b}(\omega_{ie}^{n}+\omega_{en}^{n})\;$$

(7) $$\omega_{ie}^{n}={\left[\begin{array}{ccc} 0 & \Omega \cos L & \Omega \sin L \end{array}\right]}^{T}\;$$

(8) $$\omega_{en}^{n}={\left[\begin{array}{ccc} -V_{N}/R_{M} & V_{E}/R_{N} & V_{E}/\left(R_{N}\tan L\right) \end{array}\right]}^{T}\;$$

(9) $$G^{n}={\left[\begin{array}{ccc} 0 & 0 & -g \end{array}\right]}^{T}\;$$

$C_{b}^{n}$ is the direction cosine matrix of transformation from the body frame b to the local geographic frame n, from which the attitude of the vehicle can be extracted. $\omega_{ib}^{b}$ and $f^{b}$ are the outputs of the gyroscopes and accelerometers, which represent the angular rate and specific force in frame b with respect to the inertial frame i, respectively. $V^{n}$ is the velocity in frame n, where $V_{E,N,U}$ denote the east, north and upward velocities, respectively. $\left(\omega_{nb}^{b}\times \right)$ is the skew-symmetric matrix of $\omega_{nb}^{b}$, which is the angular rate of frame b to frame n in frame b. $\omega_{ie}^{n}$ is the Earth rate vector, and $\omega_{en}^{n}$ is the angular rate vector of frame n to the Earth frame e in frame n. $G^{n}$ is the projection of the gravity vector in frame n, while Ω is the Earth’s rotation rate. $C_{n}^{b}$ is the transposed matrix of $C_{b}^{n}$. The latitude L, longitude λ and height *h* are updated according to the velocity, where $R_{M}$ and $R_{N}$ are the radii of the meridian and the prime vertical, respectively.

The relationship between $C_{b}^{n}$ and the attitude of the vehicle is:

(10) $$C_{b}^{n}=\left[\begin{array}{ccc} \cos R\cos H+\sin R\sin H\sin P & \sin H\cos P & \sin R\cos H-\cos R\sin H\sin P\\ -\cos R\sin H+\sin R\cos H\sin P & \cos H\cos P & -\sin R\sin H-\cos R\cos H\sin P\\ -\sin R\cos P & \sin P & \cos R\cos P \end{array}\right]\;$$

Let $C_{b}^{n}=\left[C_{ij}\right]\;\left(i,j=1,2,3\right)$. The pitch, roll and yaw data can be obtained as:

(11) $$\begin{array}{c} P=\sin^{-1}C_{32},\\ R=\tan^{-1}\left(-C_{13}/C_{33}\right),\\ H=\tan^{-1}\left(C_{12}/C_{22}\right). \end{array}$$

Then, the error model of the INS can be derived [25]:

(12) $${\dot{\phi }}^{n}=-(\omega_{in}^{n}\times )\phi^{n}+\delta \omega_{in}^{n}-C_{b}^{n}\varepsilon^{b}$$

(13) $${\dot{\delta V}}^{n}=-\left(\left(\delta \omega_{ie}^{n}\times \right)+\left(\delta \omega_{in}^{n}\times \right)\right)V^{n}+C_{b}^{n}\nabla^{b}+\left(\left(\omega_{ie}^{n}\times \right)+\left(\omega_{in}^{n}\times \right)\right)\delta V^{n}-\left(\phi^{n}\times \right)f^{b}$$

(14) $$\dot{\delta L=}\delta V_{N}/R_{M}$$

(15) $$\delta \dot{\lambda }=\delta V_{E}/\left(R_{N}\cos L\right)+\delta LV_{E}\tan L$$

(16) $$\dot{\delta h=}\delta V_{U}$$

where $\varepsilon^{b}$ is the gyroscope error, and $\nabla^{b}$ is the accelerometer error. $\omega_{in}^{n}=\omega_{ie}^{n}+\omega_{en}^{n}$. δ denotes the error of the corresponding parameters.

### 2.2. INS/ZUPT-Integration by KF

A 15-state KF is employed to complete the integration. The state vector X is defined as:

(17) $$X=\left[\phi_{E}\;\phi_{N}\;\phi_{U}\;\delta V_{E}\;\delta V_{N}\;\delta V_{U}\;\delta L\;\delta \lambda \;\delta h\;\nabla_{x}\;\nabla_{y}\;\nabla_{z}\;\varepsilon_{x}\;\varepsilon_{y}\;\varepsilon_{z}\right]\;$$

where $\phi_{E,N,U}$ are misalignment angles of the calculated platform in n, and $\delta V_{E,N,U}$ are east, north and upward velocity errors, respectively. δL, δλ and δh denote the latitude, longitude and height errors, respectively. $\nabla_{x,y,z}$ and $\varepsilon_{x,y,z}$ represent the accelerometer biases and gyro biases in three directions of the frame b.

The process model and observation model are:

(18) $$\{\begin{array}{c} \dot{X}=FX+GW\\ Z=HX+v \end{array}\;$$

F is the system matrix, which can be obtained according to the error model of INS. G is the system noise matrix, Z is the observation vector, H is the observation matrix, and W and v are process noise vector and observation noise vector.

When the dynamic ZUPT condition is satisfied, the lateral and vertical velocity of the body frame should be zero, and the longitudinal velocity is provided by DVL. The observation vector can be calculated as [26]:

(19) $$Z=V_{INS}^{b}-V_{ZUPT}^{b}=\left[\begin{array}{c} V_{INS,x}^{b}\\ V_{INS,y}^{b}\\ V_{INS,z}^{b} \end{array}\right]-\left[\begin{array}{c} 0\\ V_{y}^{b}\\ 0 \end{array}\right]\;$$

The relationship between the observation vector and the state vector is derived as follows:

(20) $$Z=V_{INS}^{b}-V_{ZUPT}^{b}=C_{n}^{b}C_{n^{\prime }}^{n}{\hat{V}}_{INS}^{n^{\prime }}-V_{true}^{b}+v=C_{n}^{b}\left(\phi \times \right)V^{n}+C_{n}^{b}\delta V^{n}+v\;.$$

The corresponding observation matrix can be obtained:

(21) $$H=\left[\begin{array}{ccc} A_{3\times 3} & B_{3\times 3} & 0_{3\times 9} \end{array}\right]$$

(22) $$A=\left[\begin{array}{l} C_{n}^{b}\left(1,3\right)V_{N}-C_{n}^{b}\left(1,2\right)V_{U}\;C_{n}^{b}\left(1,1\right)V_{U}-C_{n}^{b}\left(1,3\right)V_{E}\;C_{n}^{b}\left(1,2\right)V_{E}-C_{n}^{b}\left(1,1\right)V_{N}\\ C_{n}^{b}\left(2,3\right)V_{N}-C_{n}^{b}\left(2,2\right)V_{U}\;C_{n}^{b}\left(2,1\right)V_{U}-C_{n}^{b}\left(2,3\right)V_{E}\;C_{n}^{b}\left(2,2\right)V_{E}-C_{n}^{b}\left(2,1\right)V_{N}\\ C_{n}^{b}\left(3,3\right)V_{N}-C_{n}^{b}\left(3,2\right)V_{U}\;C_{n}^{b}\left(3,1\right)V_{U}-C_{n}^{b}\left(3,3\right)V_{E}\;C_{n}^{b}\left(3,2\right)V_{E}-C_{n}^{b}\left(3,1\right)V_{N} \end{array}\right]$$

(23) $$B=C_{n}^{b}$$

Equation (18) is transformed into the discrete time formula:

(24) $$\{\begin{array}{c} X_{k}=\phi_{k,k-1}X_{k-1}+G_{k}W_{k}\\ Z_{k}=H_{k}X_{k}+V_{k} \end{array}$$

The update and prediction processes are illustrated as follows [27]:

(25) $${\hat{X}}_{k,k-1}=\phi_{k,k-1}{\hat{X}}_{k-1},$$

(26) $$P_{k,k-1}=\phi_{k,k-1}P_{k-1}\phi_{k,k-1}^{T}+G_{k,k-1}Q_{k-1}G_{k,k-1}^{T}$$

(27) $$K_{k}=P_{k,k-1}H_{k}^{T}{\left[H_{k}P_{k,k-1}H_{k}^{T}+R_{k}\right]}^{-1},$$

(28) $${\hat{X}}_{k}={\hat{X}}_{k,k-1}+K_{k}\left[Z_{k}-H_{k}{\hat{X}}_{k,k-1}\right],$$

(29) $$P_{k}=\left[I-K_{k}H_{k}\right]P_{k,k-1}.$$

${\hat{X}}_{k,k-1}$ and ${\hat{X}}_{k}$ are the predicted state estimate and updated state estimate, respectively. $\phi_{k,k-1}$ is the state transition matrix, while $P_{k,k-1}$ and $P_{k}$ are the predicted estimate covariance and updated estimate covariance. $K_{k}$ is the Kalman matrix, where $Q_{k-1}$ and $R_{k}$ are the variance–covariance matrices of the states and observation, respectively.

## 3. IMM-Aided ZUPT Solution

Compared to the land vehicle, the underwater vehicle’s motion is easily influenced by the water current. When the surrounding water current remains still, applying the dynamic ZUPT solution is a proper way to mitigate the lateral and vertical velocity errors caused by the DVL velocity errors. However, when a water current exists, it is quite easy for the underwater vehicle to obtain a lateral or vertical velocity, where the ZUPT solution no longer works. Thus, the IMM algorithm is introduced into the underwater INS/DVL-integrated navigation system to ensure the capability of both INS/ZUPT and INS/DVL models in their applicable environment. Meanwhile, when DVL is unavailable, where the system is operating under the pure INS mode, the IMM algorithm is also explored to detect the ZUPT condition and make a balance between the ZUPT mode and pure INS mode.

The essence of the IMM algorithm is to obtain a weighted sum of the estimations of the ZUPT and non-ZUPT filters, which are updated in parallel. After calculating the innovation and innovation covariance matrix of each model, the IMM algorithm is able to choose the optimal model to describe the current driving situation. In prior research, the ZUPT detector was needed to identify the ZUPT driving condition, where the IMU sensor data is carefully analyzed. In contrast, the proposed IMM-aided ZUPT solution employs the innovation and innovation covariance information to autonomously identify the optimal model in the current moment, which avoids the complex identification indicated by IMU sensors and reduces the time delay.

Based on the Markovian transition probability between different models, the interactive process of the individual filters can be described in four parts [28].

(1) Interaction:

Each model has its own filter. Given the Markov model transition probability and model probability calculated at the end of the previous cycle, the state vector and the estimate covariance of each filter are updated according to the model transition probability:

(30) $$u_{i\to j}(k-1|k-1)=p_{i\to j}u_{i}\left(k-1\right)/\overset{2}{\underset{i=1}{\sum }}p_{i\to j}u_{i}\left(k-1\right)\;$$

(31) $${\hat{X}}_{oj}\left(k-1|k-1\right)=\overset{2}{\underset{i=1}{\sum }}{\hat{X}}_{i}\left(k-1|k-1\right)u_{i\to j}\left(k-1|k-1\right)\;$$

(32) $$\begin{array}{c} P_{oj}(k-1|k-1)=\overset{2}{\underset{i=1}{\sum }}\{P_{i}\left(k-1|k-1\right)+[{\hat{X}}_{i}\left(k-1|k-1\right)-{\hat{X}}_{oj}(k-1|k-1)]\\ \times {\left[{\hat{X}}_{i}\left(k-1|k-1\right)-{\hat{X}}_{oj}(k-1|k-1)\right]}^{T}\}\times u_{i\to j}(k-1|k-1) \end{array},$$

where $u_{i}\left(k-1\right)$ is the model probability updated in the last epoch. $p_{i\to j}$ is the Markov model transition probability from model *i* to model *j*. $u_{i\to j}(k-1|k-1)$ is the model transition probability. ${\hat{X}}_{oj}\left(k-1|k-1\right)$ is the estimated state vector of model *j*, and $P_{oj}\left(k-1|k-1\right)$ is the corresponding covariance.

(2) Model Filtering:

Given the interacted state vector and covariance matrix of each model, the traditional KF is operated in individual filters, which are referred to the Equations (25)–(29). The innovations and their covariance matrices should be recorded to update the model probability in the next step, which are:

(33) $$r_{j}\left(k\right)=Z_{j}\left(k\right)-H_{j}\left(k\right){\hat{X}}_{j}\left(k|k-1\right),$$

(34) $$S_{j}\left(k\right)=H_{j}\left(k\right)P_{j}\left(k|k-1\right)H_{j}^{T}\left(k\right)+R_{j}\left(k\right),$$

${\hat{X}}_{j}\left(k|k-1\right)$ and $P_{j}\left(k|k-1\right)$ are the predicted state estimate and its covariance of model *j*.

(3) Model Probability Update:

The model probability is updated according to the innovations and innovation covariance matrices. Assuming the innovation obeys the Gaussian distribution with a mean value of 0 and a variance of $S_{j}\left(k\right)$, the likelihood function is:

(35) $$f_{j}\left(k\right)=\exp\left(-\frac{1}{2}r_{j}^{T}\left(k\right)S_{j}^{-1}\left(k\right)r_{j}\left(k\right)\right)/{\left({\left(2\pi \right)}^{m}\left|S_{j}\left(k\right)\right|\right)}^{1/2}\;$$

where *m* is the dimension of the observation vector. The model probability is updated according to the different $f_{j}\left(k\right)$, Markov model transition probability $p_{i\to j}$ and previous model probability $u_{i}\left(k-1\right)$:

(36) $$u_{j}\left(k\right)=f_{j}\left(k\right)\overset{2}{\underset{i=1}{\sum }}p_{i\to j}u_{i}\left(k-1\right)/\left(\overset{2}{\underset{j=1}{\sum }}f_{j}\left(k\right)\overset{2}{\underset{i=1}{\sum }}p_{i\to j}u_{i}\left(k-1\right)\right)\;$$

(4) Output Combination:

Given the newly updated weight, the outputs of individual filters are integrated according to their different model probability.

(37) $${\hat{X}}_{k}=\overset{2}{\underset{i=1}{\sum }}u_{i}\left(k\right){\hat{X}}_{i}\left(k|k\right)\;,$$

(38) $$P_{k}=\overset{2}{\underset{i=1}{\sum }}\{P_{i}\left(k|k\right)+[{\hat{X}}_{i}\left(k|k\right)-{\hat{X}}_{k}]{\left[{\hat{X}}_{i}\left(k|k\right)-{\hat{X}}_{k}\right]}^{T}\}$$

## 4. Simulations

Simulations are conducted to evaluate the proposed IMM-aided ZUPT methodology for underwater INS/DVL-integrated navigation system. Different from the land vehicles, whose lateral and vertical velocities can be always set zero, the validity of the ZUPT solution of the underwater vehicles should be researched under the complex water current environment. The traditional underwater navigation system has no knowledge about whether there is a water current or not. Thus, the ZUPT solution, which can be easily applied to the land vehicles, cannot be directly employed in the underwater environment. The proposed IMM-aided ZUPT solution shows its advantages, which works whenever water current exists or not. First, compared to INS/DVL integration, the effectiveness of the INS/ZUPT solution is investigated when the underwater vehicle is driving under the no water current environment. Then, when the water current exists, whether the proposed algorithm can identify the non-ZUPT condition and navigate with the traditional INS/DVL solution or not is explored. Besides, the capability of the real time model estimation of the proposed algorithm when the vehicle travels under different water current conditions is evaluated through the experiments. In addition, when DVL is unavailable, the effectiveness of the prediction of whether the system should work under the pure INS mode or ZUPT mode is studied.

**Figure 1:**
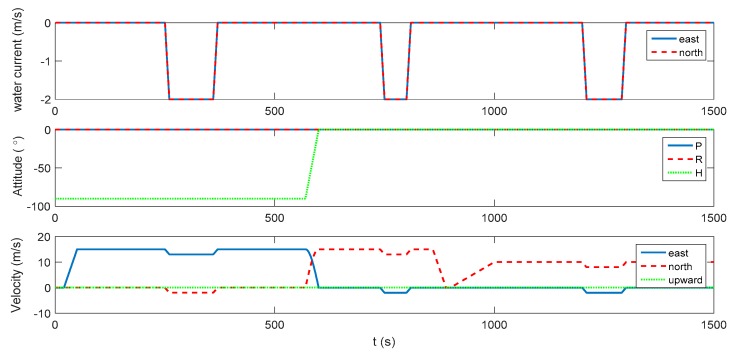
Water current and vehicle dynamics.

**Figure 2:**
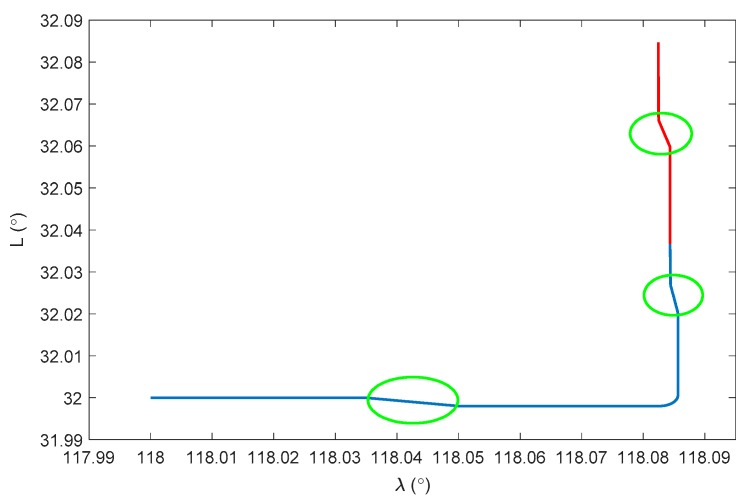
Navigation trajectory.

The whole experiment lasts for 1500 s, and consists of acceleration, deceleration, uniform motion and turning motion. The integration experiment, where DVL is available, is operated in the first 900 s. When DVL is unavailable after 900 s, the performance of the proposed algorithm is also evaluated and compared with the pure INS solution. The initial position of the carrier is set as 32° in latitude and 118° in longitude, and the initial velocity is 0 m/s. The initial heading is −90° in the east direction and the pitch and roll are set as 0°. The INS is assumed installed along the body frame of the vehicle, and the installation error between DVL and INS is assumed well-compensated off line. The update rate of the IMU is 200 Hz and the DVL velocity is outputted at 1 Hz. The constant biases and random noises of the gyroscopes are all set as 0.01 °/h, while those of the accelerometers are set as 50 μg. This is a simplified assumption of the fiber-optic IMUs, where sensor’s coupling coincident scale factors, installing error, system error should also exist in real applications. However, these errors can be calculated exactly and compensated by a calibration test, which is always conducted before the real tests [29]. Thus, for simplicity, the above-mentioned errors will not be discussed any further here, as they are not the focus of this research. Due to the complex underwater environment, there are always errors in the DVL velocity. To simplify the simulation, a 0.03 m/s constant velocity error and a 0.02 m/s random noise are assumed in the DVL velocity throughout the whole experiment, which is a medium level of DVL accuracy to evaluate the universality of the proposed algorithm. A depthometer is involved to measure the height information, which is the most common sensor in the underwater navigation system. The water current is assumed static at the beginning. From 250 s, there is assumed to be a water current towards the southwest direction, which reaches 2.828 m/s. The current vanishes at 370 s and starts again from 740 s to 810 s and from 1200 s to 1300 s. The velocity of the water current and the vehicle dynamics are shown Figure 1. The trajectory is presented in Figure 2.

**Figure 3:**
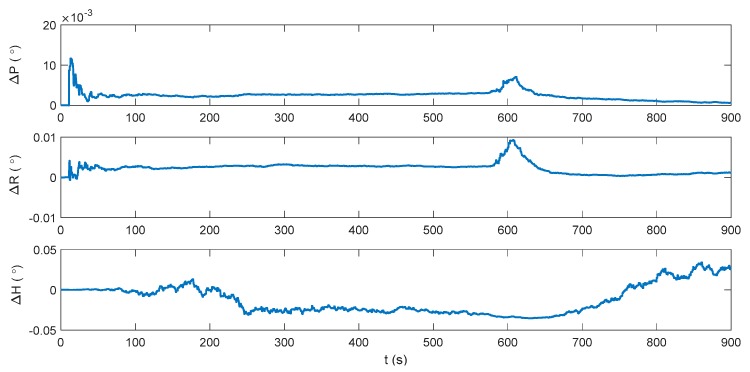
Attitude error of the inertial navigation system/Doppler velocity log (INS/DVL)-integrated navigation system.

**Figure 4:**
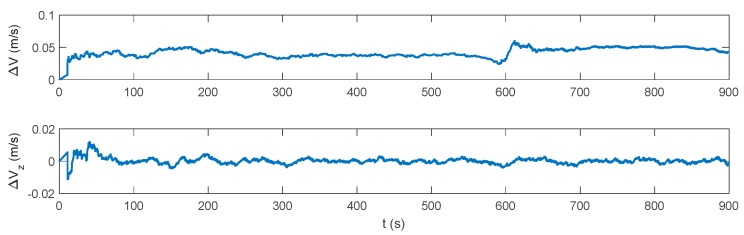
Velocity error of the INS/DVL-integrated navigation system.

**Figure 5:**
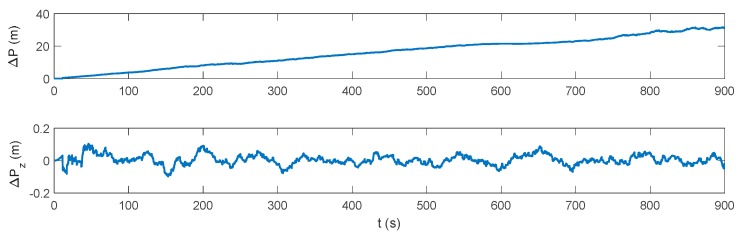
Position error of the INS/DVL-integrated navigation system.

The blue line in Figure 2 denotes the integration period, while the red line indicates the DVL outage period. Marked with green ellipses in Figure 2, three periods of water current are involved in the whole voyage, two of which occur during the integration period. It can be seen that during the three periods, the heading of the underwater vehicle is assumed stable, where only the velocity is influenced by the water current. Thus, the lateral velocity will be introduced during these three periods, which is always zero on other occasions. Firstly, the performance of the traditional INS/DVL integration is shown in Figure 3, Figure 4 and Figure 5, where the errors of the pitch, roll, yaw, horizontal velocity, vertical velocity, horizontal position and vertical position are shown successively.

**Figure 6:**
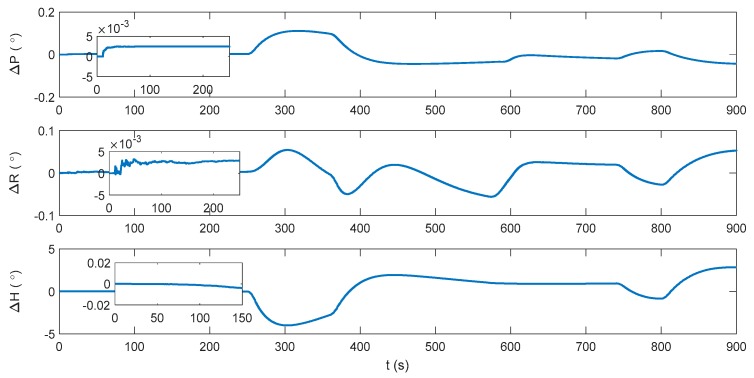
Attitude error of the INS/zero velocity update (ZUPT) solution.

**Figure 7:**
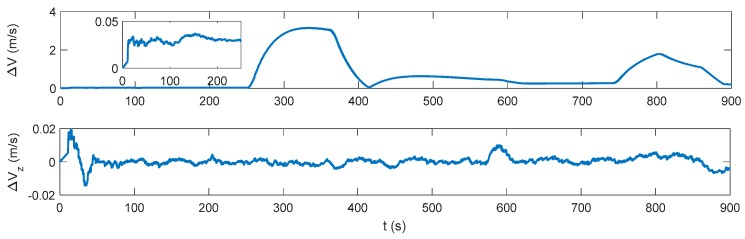
Velocity error of the INS/ZUPT solution.

**Figure 8:**
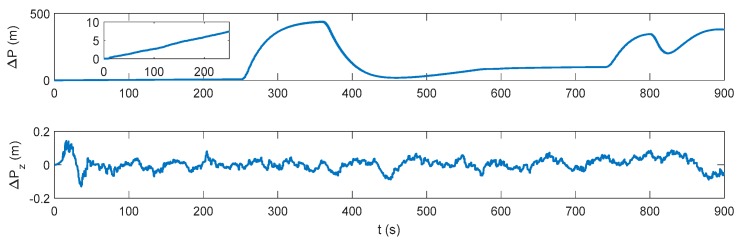
Position error of the INS/ZUPT solution.

It can be seen that the attitude error is below 0.005°, 0.005° and 0.05° in pitch, roll, and yaw, respectively. Due to the erroneous velocity information provided by DVL, the mean value of the horizontal velocity error is 0.04149 m/s. The vertical velocity is only about 0.01 m/s because a depthometer is involved in the system to restrict the vertical errors. The horizontal position error increases monotonically with time, reaching 31.22 m at 900 s, and the vertical positioning error is within 0.2 m. In an INS/DVL underwater navigation system, where the external velocity data forms the observation vector, rather than the position information, the positioning error would accumulate over time without the acquirement of the external position information. Any error suppression method is significant to the system, as the positioning error in each moment will be introduced to the next moment. Thus, the dynamic ZUPT solution is investigated to mitigate the lateral velocity error and decrease the accumulation of the position error. Its performance is shown in Figure 6, Figure 7 and Figure 8.

From 0 s to 250 s, where no water current exists, the attitude performance of the INS/ZUPT solution is similar to that of the INS/DVL solution. However, as the lateral velocity is bounded by the ZUPT condition, the horizontal velocity error of the INS/ZUPT solution is smaller, at around 0.0313 m/s. As a result, the horizontal position error of the INS/ZUPT solution is 7.345 m at 250 s, while that of the INS/DVL solution reaches 9.108 m. Thus, the INS/ZUPT outperforms the INS/DVL solution under the no water current environment. However, when the water current occurs at 250 s, the lateral velocity of the vehicle is no longer zero. The erroneous restriction on the lateral velocity of the vehicle collapses the whole system, where the attitude, velocity and position errors run within 400 s up to 4°, 3.112 m/s and 438.6 m, respectively. Therefore, the proposed IMM-aided ZUPT methodology for INS/DVL integration is studied to leverage the strength of both the INS/ZUPT and INS/DVL systems. The initial model probabilities are 0.9 and 0.1 for INS/DVL and INS/ZUPT models, respectively.

**Figure 9:**
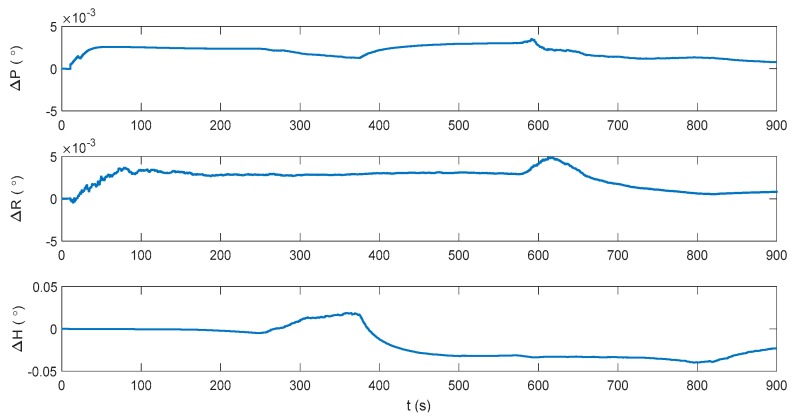
Attitude error of the interactive multiple model (IMM)-aided ZUPT solution.

**Figure 10:**
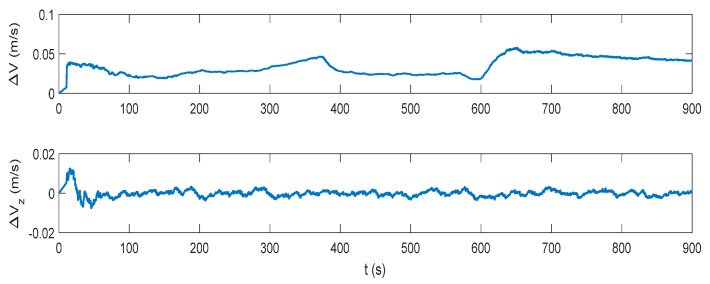
Velocity error of the IMM-aided ZUPT solution.

**Figure 11:**
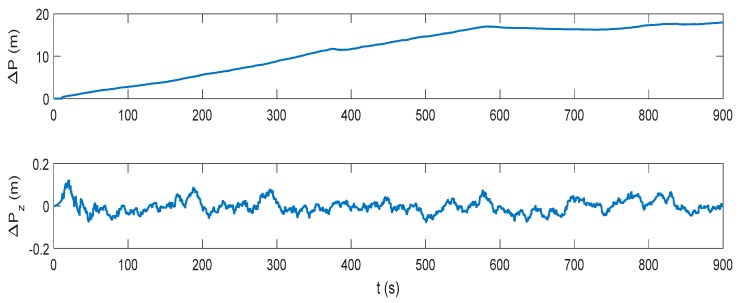
Position error of the IMM-aided ZUPT solution.

**Figure 12:**
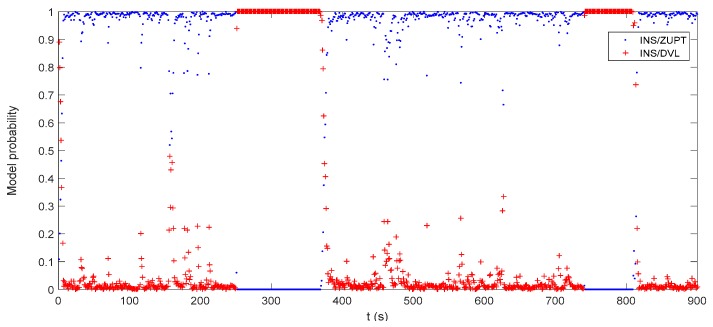
Model probability of the IMM-aided ZUPT solution.

The attitude, velocity and position errors of the proposed algorithm are shown in Figure 9, Figure 10 and Figure 11. It can be seen that the proposed method outperforms both the INS/DVL and INS/ZUPT solutions. When the water current does not exist, the proposed algorithm shows the similar navigation accuracy as the INS/ZUPT method. When the water current occurs, the proposed algorithm can identify the situation rapidly and switch the system into non-ZUPT mode. The mean value of the horizontal velocity error is 0.03387 m/s for the IMM-aided ZUPT solution, which is smaller than the INS/DVL solution. At 900 s, the position error of the proposed solution is 17.98 m, which is only about 57% of the position error of the INS/DVL method. The attitude performance is similar to that of the INS/DVL solution. Figure 12 shows the model switching process, where the blue dots indicate the model probability of INS/ZUPT and red crosses denote the model probability of INS/DVL. It is obvious that the proposed algorithm can clearly identify the proper model for the present moment. When the water current occurs, the proposed system can figure out the situation without any delays. When the water current vanishes, the proposed system will return to the INS/ZUPT model in 5 s. Since the false selection of the INS/ZUPT model is much more serious than that of the INS/DVL model according to Figure 3, Figure 4, Figure 5, Figure 6, Figure 7 and Figure 8, the proposed algorithm is fault-tolerant and sensitive to the non-ZUPT mode, and is reserved to the ZUPT mode.

It can be concluded that the INS/ZUPT method can mitigate the velocity and position errors when the underwater vehicle travels with no water current, but fail to navigate when the water current exists. The proposed IMM-aided ZUPT methodology outperforms both the INS/DVL and INS/ZUPT methods, which can easily identify the ZUPT and non-ZUPT condition and make a leverage between the two models. By analyzing the innovations and innovation covariance matrices, the IMM algorithm can automatically allocate the corresponding weights for the two models.

Furthermore, when DVL is unavailable, the capability of the proposed algorithm is also evaluated. Normally, the IMM algorithm is only used in the integrated system. In this work, the IMM algorithm is also employed during DVL outage to identify the proper solution for the standalone INS. The whole DVL outage period starts from 900 s and ends at 1500 s. The proposed IMM-aided ZUPT algorithm is utilized to navigate from 0 s to 900 s, which have shown the best performance during the integration period. A 100 s water current is assumed from 1200 s to 1300 s.

**Figure 13:**
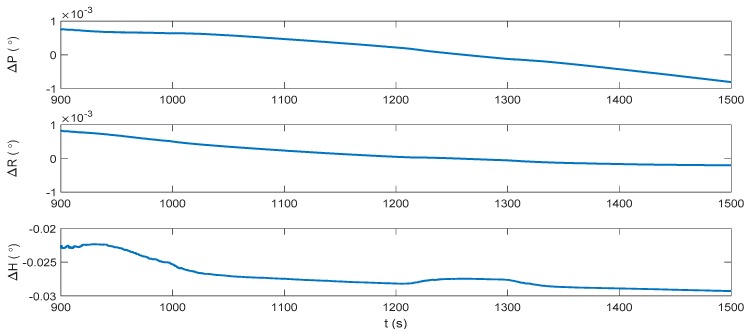
Attitude error of the pure INS solution during the DVL outage.

**Figure 14:**
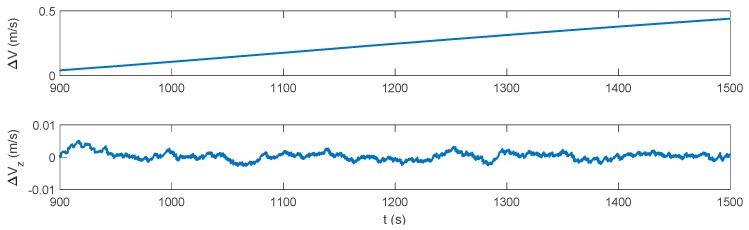
Velocity error of the pure INS solution during the DVL outage.

**Figure 15:**
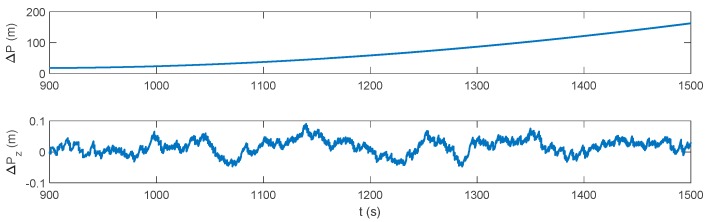
Position error of the pure INS solution during the DVL outage.

Figure 13, Figure 14 and Figure 15 show the attitude, velocity and position errors of the system under the pure INS mode during the DVL outage. It can be seen that without DVL, the horizontal velocity error increases immediately, and reaches 0.439 m/s at 1500 s. As a result, the horizontal position error accumulates to 162.2 m in the end. ZUPT is the most common algorithm for the land vehicles to mitigate the error divergence, which could reduce the accumulation of the velocity and position errors in the lateral direction of the vehicle. However, for the underwater vehicles, where the zero velocity condition is easily unsatisfied, the ZUPT solution should be used carefully to avoid the divergence of the navigation errors. Similar to the INS/DVL integration period, the direct usage of ZUPT will cause a failure during the DVL outage period, and it will not be further discussed.

**Figure 16:**
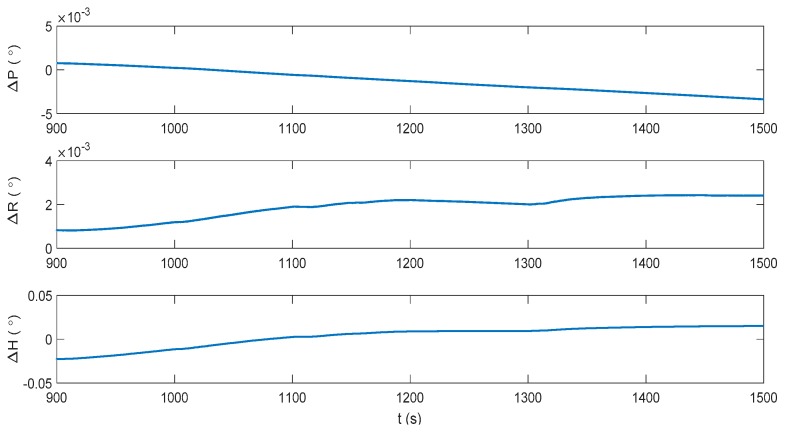
Attitude error of the IMM-aided ZUPT solution during the DVL outage.

**Figure 17:**
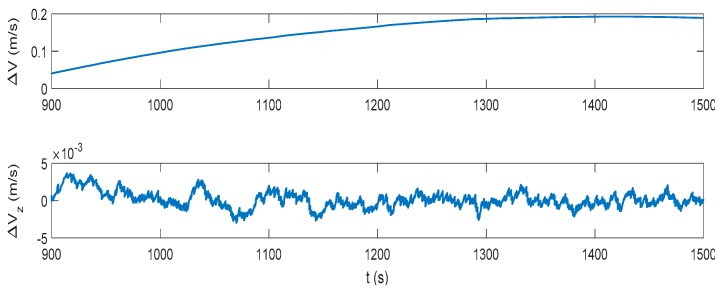
Velocity error of the IMM-aided ZUPT solution during the DVL outage.

**Figure 18:**
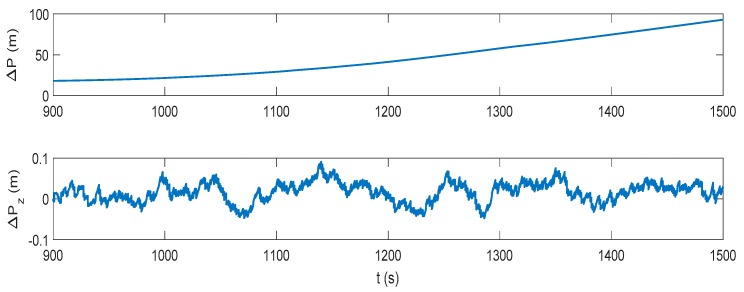
Position error of the IMM-aided ZUPT solution during the DVL outage.

**Figure 19:**
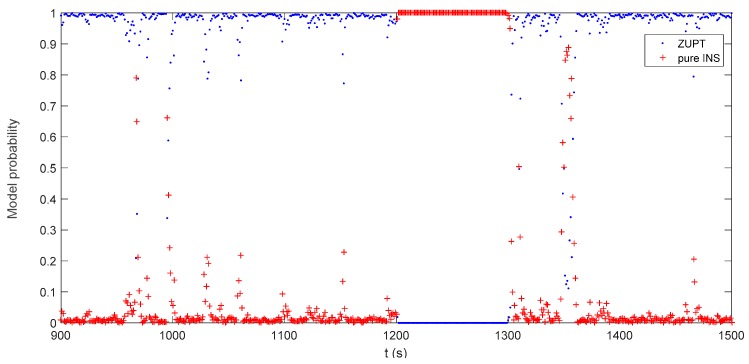
Model probability of the IMM-aided ZUPT solution during DVL outage.

The IMM-aided ZUPT solution is evaluated during DVL outage. The observation vector of the former INS/DVL model is set zero to represent the pure INS model. The longitudinal velocity observation element of former INS/ZUPT model is set zero as no DVL velocity is provided, while the observation element of the lateral velocity remains the difference between the lateral velocity calculated by INS and the zero velocity. The performance of the proposed algorithm during the DVL outage is shown in Figure 16, Figure 17, Figure 18 and Figure 19. Compared to the pure INS mode, the proposed IMM-aided ZUPT solution can largely restrain the divergence of the velocity and position errors and improve the navigation accuracy. The velocity error is about 0.2 m/s at the end of the experiment, while the position error is 92.93 m after the 600 s DVL outage. The attitude error is similar to that of the pure INS mode. The model probability is shown in Figure 19. It can be seen that the proposed algorithm is also able to identify the proper model at the present moment when DVL is unavailable. Compared to the existing ZUPT detecting algorithms for the standalone INS, the proposed IMM-aided ZUPT solution can successfully identify the ZUPT condition and employ the ZUPT solution properly simultaneously and autonomously. Its structure remains the same for both the INS/DVL integration and standalone INS, which is easy to switch when DVL outage occurs.

## 5. Conclusions

In this work, an IMM-aided ZUPT algorithm is proposed for INS/DVL integrated navigation system to take the advantages of both ZUPT method and INS/DVL traditional method under the complex underwater environment. Due to the unstable temperature, water density and salinity, the residual errors in the DVL velocity data will influence navigation accuracy of the underwater vehicles. To reduce the negative effect of the DVL velocity error on the whole system, the ZUPT solution is introduced to mitigate the lateral velocity and position errors. However, as a water current often exists, the performance of the ZUPT method would be largely influenced. Thus, IMM algorithm is introduced to form a novel IMM-aided ZUPT methodology to deal with the complex underwater navigation situations.

In the proposed algorithm, both INS/DVL model and INS/ZUPT model are constructed and operated in parallel. Through the IMM algorithm, the weights of the two model are calculated according to their innovations and innovation covariance matrices. Thus, the system can employ the two models properly and simultaneously. Meanwhile, the proposed IMM-aided ZUPT solution can also be used when DVL information is unavailable. By setting the corresponding elements of the observation vector to zero, the INS/DVL model turns into the pure INS model, while the ZUPT model for the integration turns into the ZUPT model for the standalone INS. The proposed methodology can continuously improve the navigation accuracy whenever DVL is available.

Simulations are conducted to evaluate the proposed algorithm under both the integration period and standalone INS period. It can be seen that the proposed algorithm can leverage the strength of both the INS/DVL model and INS/ZUPT model. It is also effective during DVL outage. The proper model can be clearly seen by the model probability, where the non-ZUPT period can be clearly identified for both the integration period and standalone INS period. To conclude, in the complex underwater environment, where the water current often occurs, the proposed algorithm innovatively employs the ZUPT solution using the IMM algorithm, which is designed and is suitable for the land vehicle and ineffective when lateral velocity exists. From the simulation results, it can be seen that the solution will relieve the difficulties of the underwater navigation to some extent. In the future, the field tests need to be further carried out to validate this technique in various trajectories in the real underwater environment.
